# The Investment of Funds

**Published:** 1914-01-10

**Authors:** 


### NATIONAL INSURANCE ACT DEVELOPMENTS.

### The Investment of Funds.

Attention has for some time past been so much concentrated upon matters incidental to the collection of contributions and the payment and administration of the benefits of the Act, that little or no regard has been paid by the public at large to the fact that a considerable sum is now available for permanent investment by, or on behalf of, the approved societies. It, perhaps, hardly needs demonstration, though it is a point that very frequently escapes notice, that the careful choice of investments in which to place the funds of the approved societies and the judicious supervision of these investments once they are made, is just as vital to the well-being of the societies as a whole, and of the individual members composing them, as is the care that must be taken to see that the sickness claims and claims for other benefits are not so heavy as to exceed their estimated cost.

It is essential to remember that the actuaries when estimating the contributions to be charged for the benefits of the Act, assumed in their calculations that interest would be earned on the funds at the rate of 3 per cent. per annum, and it can thus easily be seen that, other things being equal, the approved societies will only be in a sound financial position provided this rate of 3 per cent. be realised. With regard to this we would emphasise that the rate of 3 per cent. per annum required is an absolutely net rate—that is to say, 3 per cent. interest must be realised after all charges, such as income-tax and expenses of investment, have been met. Not only so, but this net rate must be obtained over the whole fund and must, moreover, be earned without any capital loss on the investments. The retention by the societies of any large sum uninvested will thus necessitate the realisation of a higher rate of interest than 3 per cent. on the remainder of the funds, and this point, together with the careful provision that must be made to guard against the depreciation of securities, will require the close attention of those who control the societies' financial affairs.

### The Highest Interest with Security.

It has been laid down in connection with the investment of the funds of life assurance companies, and the canons apply with equal force to the investment of the funds with which we are now dealing, (1) that the first consideration should invariably be the security of the capital; (2) that the highest practicable rate of interest be obtained, but that this principle should always be subordinate to the previous one, the security of the capital. These two canons may appear axiomatic, but they are capable of very divergent interpretations.

The general importance of the first canon has already been pointed out, and the value of the second will be readily appreciated when it is borne in mind that the realisation of a higher net rate of interest than 3 per cent. by any approved society over the whole of its funds would go a long way to counterbalance losses in other directions, even if it did not enable the society in course of time to provide increased benefits.

Long experience has taught those responsible for the investments of the large assurance companies that the " security of the capital " must be considered with reference to the investments as a whole, and that to limit the investments to a few particular securities, good though these securities may be, is a fatal policy if the best results are desired.

### Spreading of Investments.

It is becoming increasingly realised that the only way to obtain a good return on the money invested, and at the same time to retain " security of capital " in the truest sense, is judiciously to spread the investments over a wide area, both with regard to geographical position and type of security, so that any depreciation in one quarter is balanced by appreciation in another. If, however, the text of the Act be any guide to the intentions of the Government, the policy advocated and enforced by them is almost the exact opposite to that which has just been outlined, since by the Act the securities in which approved societies may invest such funds as they control, are strictly limited and consist mainly of Trustee Stocks. If persisted in, the effect of this limitation will be to create an artificial market for these stocks, in addition to that already existing, with the inevitable result that the interest yield will be lower than that of investments equally secure but not earmarked. The one channel through which the necessary improvement may possibly come is contained in the words " such other securities as may for the time being be approved by the Insurance Commissioners," and we are very glad to note that a committee has recently been appointed fully to consider this matter.

Considerable foresight has been exercised in the selection of the members of this committee, and we are pleased to notice among those chosen such prominent men in the assurance and financial world as Mr. J. A. Cook, General Manager of the Scottish Union and National Insurance Company; Mr. E. Roger Owen, General Manager of the Commercial Union Assurance Company; and Mr. W. G. Turpin, Secretary and Comptroller-General to the National Debt Commissioners. This committee, although its functions are purely advisory, has a most important task to fulfil, and the inclusion of members who are in daily contact with the practice of Insurance Companies in the selection of permanent investments augurs well for the soundness of the proposals that are likely to be made. It does not follow that the Insurance Commissioners will necessarily act on the report of the committee, but it is to be hoped that the findings will be published.

So much, then, for the future; but what of the present? What of the funds that are already in the hands of the Commissioners? Is full advantage being taken of the present state of the stock market to secure a good return on the capital invested? It would not appear so.

### The Funds Available for Investment.

The actual cash that is available for investment arises from two sources: (1) the normal excess of contributions over benefit payments; (2) the amounts periodically written off the "reserves" by the application of a specific proportion of the contributions reserved for that purpose. And it is the duty of the Commissioners, from time to time, to ascertain what sums standing to the credit of the approved societies are available for permanent investment. Of the amount thus ascertained the society is given control of four-sevenths in so far as the amount arises from the contributions of men, and one-half in so far as it arises from the contributions of women, and the balance is retained by the Commissioners for investment.

The Commissioners are empowered to hand over any sums temporarily in their charge to the National Debt Commissioners, who allow interest at a fixed rate. A fixed rate of interest is also allowed by the Insurance Commissioners on the sums to be invested by them in permanent investments, and this rate is at present, in both cases, 3¼ per cent. per annum.

Bearing in mind the importance to approved societies of the rate of interest earned, we would ask, Is 3¼ per cent. the highest practicable rate that can be allowed at the present time? We are, of course, fully aware that any loss from depreciation will fall on the Commissioners, and that the expenses of investment have to be met, but it must also be recognised that the present moment is a peculiarly favourable one for investment in practically all the stock markets, owing to the recent heavy fall in prices.

### The Present Opportunity.

The truth of this last statement may perhaps be demonstrated to the best advantage by considering the interest yield obtained on investments in one or two representative groups of securities, and for present purposes we will confine ourselves to the limits laid down by the Act, although within these limits the general interest yield is somewhat below that of the market as a whole.

1. Consols.—Owing to various special reasons there is always a demand for Consols, which keeps the interest yield permanently below the average, and yet at the present moment Consols may be purchased for about 72, yielding a return as high as £3 9s. per cent. per annum.

2. India Stocks.—Any one of the three India stocks may be purchased to yield very nearly 4 per cent., the middle market prices having recently been: India 3½ per Cent. Stock, 85¼; India 3 per Cent. Stock, 72⅝; India 2½ per Cent. Stock, 61.

3. Colonial Government Stocks.—These may be purchased at the present moment to yield anything from 4 per cent. to 4½ per cent., with ample security.

4. Home Railway Debenture Stocks.—These stocks, which afford absolute security, may also be purchased to yield between 4 per cent. and 4½ per cent.

So the list might be extended. The point that we desire to emphasise is that at the present time a rate of interest of over 4 per cent. may comfortably be earned even within the limits laid down by the Act, and we submit that if full advantage were taken of this state of affairs a higher rate than 3¼ per cent. could well be allowed to approved societies. A margin of over ¾ per cent. should be more than sufficient to provide for any likely depreciation and to cover all the ordinary expenses of investment.

If it is the intention of the Government always to keep the rate allowed so much below that which could be earned, it behoves approved societies to take into their own hands the task of investing as much of their funds as is permitted by the Act. It must be borne in mind that up to the present only a small proportion of such funds have been handed over, and the main interest earned, therefore, has been that allowed by the Commissioners; it must furthermore be noticed that even this rate of 3¼ per cent. has been received on only a comparatively small portion of the total funds, since at present the much larger proportion is composed of the book debt consisting of the "reserves" on which interest is only allowed at the minimum rate which is necessary to ensure solvency—namely, 3 per cent. Over the whole funds, therefore, interest received up to the present has been only slightly over 3 per cent., and there is thus little hope that much will be contributed from this source to the relief of approved societies at the first valuation.

### An Anomaly for the Government.

It is somewhat interesting to note that it is indirectly to the advantage of the Government that the rate of interest allowed by the Commissioners to approved societies be kept as low as possible. The Government in contributing their share to the cost of the benefits, do so, not by way of an addition to the contributions, but by paying a proportion of the cost of the benefits.

Now a high rate of interest earned on the funds results, other things being equal, in the realisation of a surplus and a consequent increase in benefits, and an increase in benefits brings with it a greater contribution from the Government. Even if a surplus be not realised at least a deficiency may be prevented, and in either case it is obviously to the advantage of the Government to keep the rate of interest low rather than high. It is not suggested that considerations such as these would influence the Commissioners in deciding upon the rate to be allowed, but it is well to bear clearly in mind that the interests of the Government are directly opposed to those of the approved societies on this point.

---

NOTE.—Questions and Correspondence on all doubtful points are invited, and should be addressed to the Insurance Editor, The Hospital, 28 Southampton Street, Strand.

